# European Green Deal – legal and financial challenges of the climate change

**DOI:** 10.1007/s12027-020-00637-3

**Published:** 2020-11-03

**Authors:** Alicja Sikora

**Affiliations:** 1 Jagiellonian University, Cracow, Poland; 2 UCLouvain Saint-Louis, CEDRE (Centre d’étude du droit de l’environnement), Brussels, Belgium

**Keywords:** Climate change, European Green Deal, European Green Deal Investment Plan, Constitutional dimension of environmental protection, Sustainable finance, Green transition

## Abstract

The European Green Deal announced by the European Commission in December 2019 is a roadmap meant to foster the transition of the European Union towards the climate-neutral economy by reducing carbon emissions towards 55% by 2030 and achieving carbon neutrality by 2050. By putting the EGD in a boarder perspective of evolving, constitutional rationale of environmental protection in the EU legal order, this contribution examines horizontal, legal dimension and financial implications of the green transition. The challenge ahead of the Union is now how to transform the ambitious climate agenda into efficient legal and economic instruments ‘in a fair way, leaving no one behind’. This paper argues that EGD is a great opportunity, but in order to turn it into a success, it must be strongly anchored in the concepts pertaining to the constitutional framework of the EU legal order, in particular, the concepts of solidarity, sustainable development and high level of environmental protection.

## Introduction

In April 2020, on the 50th anniversary of the Earth Day, the New York Times has published a list of inspiring books unwrapping the concept of a climate change, among which scientific, fantasy and fiction.^1^ Yet, in case of the climate change, fiction is surprisingly becoming reality. Available scientific data is pitilessly facing the political and economic global agenda with a need of a transversal action.^2^ Environmental and ecological questions have penetrated our everyday life from access to food, water and sanitation, through the use of sustainable energy and waste policy, via gaming industry to the threats to biodiversity. The schoolstrike of Greta Thunberg and Extinction Rebellion have become, in parallel, a symbol of unfulfilled concerns of the young generation.^3^ Recently, commitment to the green transition has been heralded as one of the major components in order to address the economic crisis due to the COVID-19 pandemic.^4^ The European Council in July 2020 concluded on the new scope of the climate ambitions of the European Union (EU).^5^ Indeed, “today, climate change and the Holocene extinction have emerged as the issues requiring urgent action in order to save our planet.”^6^

Against this background, the European Green Deal (EGD) announced by the European Commission (Commission) in December 2019^7^ amounts to an unprecedented attempt at the level of the Union to foster the transition towards the common goal of a climate-neutral economy by reducing carbon emissions by at least 50% by 2030 (and towards 55%) and achieving carbon neutrality by 2050. The Commission construed its work programme through the concept of environmental re-orientation of EU activities in areas identified as leading actions of the EGD Communication such as climate ambition, clean affordable and secure energy, industrial strategy for a clean and circular economy, sustainable and smart mobility, agriculture and fisheries, biodiversity, zero pollution and toxic-free environment, mainstreaming sustainability, trade and foreign policy and the European Climate Pact.^8^ Based on the concepts of sustainability and protection of the EU’s natural capital, combined with legal and financial discipline, it is meant to transform the EU by 2050 to the state “fair and prosperous society, with a modern, resource efficient and competitive economy”.^9^

EGD is rightly viewed as an *opportunity* for a change of a horizontal regulatory framework based on effective and efficient instruments both in a long and in a short term.^10^ This opportunity arises from a number of legal developments and political commitments which have made the climate law a part of the EU legal order, as formally heralded in the Lisbon treaty 11 following substantive steps of international law such as Paris Agreement,^12^ UN 2030 Agenda^13^ and the sustainable developments goals.^14^

The EGD has been followed in January 2020 the European Green Deal Investment Plan (EGDIP), which is the “investment pillar” of the EGD.^15^ EGDIP was meant to ‘mobilise at least €1 trillion of sustainable investments over the next decade’ since the intention of the Commission was to ‘enable a framework to facilitate public and private investments needed for the transition to a climate-neutral, green, competitive and inclusive economy’.^16^ As rightly emphasized in the literature, the EGD may deeply and successfully change the Union’s economy essentially if it remains a real policy priority in both the short and the long run, in particular concerning the climate agenda at the EU and global level, and if its implementation triggers substantiated reorientation of financial measures and fund allocation.^17^ Furthermore, the EGD is to be aligned with a new industrial strategy adopted on 10 March 2020, whereby the Union is expected to become a world leader in the circular economy and clean technologies, and to decarbonise energy-intensive industries.^18^

In order to grasp better the impact of the EGD, one should broaden the perspective of its assessment. Indeed, given its scope of ambition, the EGD can be analysed at various levels. First, EGD should be approached from the constitutional EU law perspective. Given its mission the EGD could represent an innovative tool allowing environmental ambitions permeate the EU legal order which requires global, constitutional thinking. In particular, since EGD addresses sustainability, it should be analysed from both environmental, economic and budgetary dimension as well as social dimension of the European project. Secondly, at the implementation level, EGD and the new climate targets are liable to perform a particular climate spill-over effect affecting the substance of the EU policies, notably the cohesion policy, State aid, emissions trading, taxation, agriculture, energy and transport, biodiversity, consumer protection and many other fields of EU action. Likewise, EGD must be linked with the financial aspects of the public and private sectors’ missions and expectations in the process of attaining the climate neutrality. Thirdly, once the EGD objectives translated into the legal instruments, one should not lose out of sight the enforcement level, in particular, the issue of judicial enforceability of environmental commitments expressed therein. In this context, judicial protection and enforcement of environmental procedural and substantive rights and to the certain extent environmental principles might be enhanced by the EGD rationale. Indeed, if attaining climate neutrality by 2050 and ensuring that all EU policies contribute to that climate neutrality remains merely an objective without efficient legal instruments of enforcement, its impact is compromised since the very beginning. In this context, it may be useful to recall the groundbreaking interpretation of the Water Framework Directive (WFD)^19^ and the objectives lied therein. The Court held that “the Member States are required — unless a derogation is granted — to refuse authorization for an individual project where it may cause a deterioration of the status of a body of surface water or where it jeopardises the attainment of good surface water status or of good ecological potential and good surfacewater chemical status by the date laid down by the directive”.^20^ This telling example which has turned a general objective of an EU directive into the substantive obligation could be relied on in the process of attainment of the climate neutrality.

In relation to environmental regulatory instruments, it is interesting to see whether the EGD is going to reinforce the integrated approach and to consider the interferences of different environmental media. Likewise, one could ask what is the EGD potential in relation to environmental procedural standards or whether it rather focuses on the substantive environmental provisions?

The title of this contribution could appear overambitious for a text which aims at exploring the intricacies and the technicalities of the EGD. One should however keep in mind that the exercise of mapping horizontal legal dimension and financial implications of the green transition is a crucial step towards better understanding of the multidimensional relevance of environmental protection in the current state of the development of the Union. This paper argues that EGD is a great opportunity, but in order to turn it into a success, it must be strongly anchored in the concepts pertaining to the constitutional framework of the EU legal order, in particular, the concepts of solidarity, sustainable development and high level of environmental protection.

## Legal dimension of the EGD – technical versus constitutional

### Environmental protection in the EU legal order – constant evolution

As a concept, ‘environmental protection does not bear a univocal definition. Neither it is limited to one specific area of regulation’.^21^ Its essence equals in proliferation and heterogeneity of the areas of concern.^22^ A general account of various concepts confirms ‘an inherent lack of certainty about what the “environment” entails and how meaningful concept of the environment’ can be translated into the legislation and adjudication.^23^ In the EU, one of the most holistic definitions of the concept of “environment” has been proposed by the European Commission in 1971 in its first environmental communication whereby environment ‘means all the elements which, interacting in a complex fashion, shape the world in which we live and move and have our being’.^24^ By offering new the growth strategy in the EGD almost 50 years later, the Commission relies in the EGD on the similar, global and prospective vision of an environmentally driven need of a change of the Union.

Historically, in the EU legal order, environmental protection equals transformation and evolution.^25^ In the last decades, EU environmental law has evolved from a sectoral, technical policy to one of guiding components of the EU legal and political substratum.^26^ Anchored in the principles of integration and sustainable development, coupled with the concept of a high level of environmental protection carved in the EU treaties, environmental protection has become an all-present and influential aspect of the EU law. The Court of Justice of the European Union (CJEU) has played a particular role in the process of this evolution and recognition. As remarked by Advocate General Jacobs about the ADBHU judgment of 1986:^27^ the CJEU ‘without any explicit legal basis – and in a context of a treaty, one of whose main aims was the elimination of trade barriers – declared environmental protection to be one of the essential objectives of the Community (…). Contrary to the recognition of fundamental rights as a general principle of Community law, the Court did not deem it necessary to justify the introduction of such an essential objective (…) by reference to any external source or support (such as national constitutional traditions or international law).’^28^

At the environmental governance level, scholars identify various stages of the development such as ‘environment regime’ which starts in 1972; the ‘internal market regime’ initiated in 1982; the ‘integration regime’ which dates back to 1992; the ‘sustainable development regime’ inaugurated in 1998.^29^ The new, ecological, climate change related stage of the development of the EU environmental law and policy is happening now. Indeed, far from considering environmental protection as a super-value or trumping principle^30^ or expression of a climate-based authoritarian state,^31^ far from considering the EU climate policy model as the most suitable at the global level,^32^ the EGD itself is a demonstration that environmental protection and climate change have become a ‘prerequisite of the further evolution of the Union’.^33^

At the level of the EU constitutional dimension, EU legal order is driven by an evolutive concept of high level of protection and improvement of the quality of the environment. Among the EU’s objectives the concept of sustainability can be considered as permeating the whole of the EU legislation.^34^ High level of environmental protection itself has been changing its legal status from an EU objective enshrined in Article 3 TEU and an underlying principle of the EU environmental law,^35^ to the transversal concept of the EU legal order interpreted jointly on the basis of Article 3 TEU, Article 191(2) TFEU and Article 37 of the Charter of Fundamental Rights. High level of environmental protection seen as a level of EU ambition^36^ is now considered as a new legal avenue for the environmental re-orientation of the EU legal order.^37^ In his Opinion in case *Bois de Vielsam*, Advocate General Bot noted that the double legal basis of high level of environmental protection raises its status to the *EU target*.^38^ In her consecutive Opinions, Advocate General Sharpston observed that “following the entry into force of the Treaty of Lisbon (…) *the principle* of “a high level of environmental protection and improvement of the quality of the environment” set out in Article 3(3) TEU and Article 37 of the Charter has become *a guiding objective of EU law*.^39^ Ultimately, Advocate General Kokott proposed to interpret provisions of the EU primary law enshrining the high level of environmental protection “are not to be interpreted or examined independently of each other. On the contrary, they give expression to the *common principle of a high level of environmental protection*, to which particular importance must be attached given the number of provisions of EU law in which it is enshrined”.^40^ In her recent Opinion in case *Craeynest*, Advocate General Kokott has equally emphasized the triple legal foundations of the emerging principle of a high level of environmental protection.^41^ Interestingly, in its judgment in case *Craeynest*, the Court ruled that the EU legislation on ambient protection “*put into concrete terms the EU’s obligations concerning environmental protection* and protection of public health, which stem, inter alia, from Article 3(3) TEU and Article 191(1) and (2) TFEU”.^42^

Finally, one should emphasize the relevance of the principle of solidarity in environmental context, in particular, in view of attaining the revolutionary climate neutrality at the EU level. Indeed, in its widest sense, solidarity lies at the foundations of the Union, constitutes even the ‘quintessence of what is both the raison d’être and the objective of the European project’.^43^ In the EU secondary law, balancing considerations of fairness and solidarity is notably referred to in the efforts sharing Regulation.^44^

### The EGD as an ambitious policy and yet technical measure

The number of contributions describing and analysing the EGD Communication of the European Commission reflects the interest sparkled by EGD.^45^ First and foremost, EGD is intended as: “a *roadmap*, a *strategy* for making the EU’s economy sustainable, becoming the first climate-neutral continent, *by proposing* a number of measures to reduce the greenhouse gas emissions (GHG) and increasing biodiversity *turning* climate and environmental challenges into opportunities across all policy areas and making the transition just and inclusive for all.” Likewise, EGD is viewed by the Commission as a “*framework of regulation and legislation* setting clear overarching targets – a blocwide goal of net zero carbon emissions by 2050, and a 50%-55% cut in emissions by 2030 (compared with 1990 levels) are supposed to be at the core – alongside incentives to encourage private sector investment, with action plans for key sectors and goals such as halting species loss, cutting waste and better use of natural resources.”^46^ Five major priorities of the EGD have been considered as most relevant, among which energy efficiency, decarbonization, just transformation towards the prosperous economy and effective climate diplomacy.^47^

The policy perspective put aside, in purely legal terms EGD is the Commission’s communication, namely a policy instrument or an instrument of the EU *soft-law*. In various fields of the EU competence, for reasons of transparency, and in order to ensure equal treatment and legal certainty, the Commission may publish acts of ‘soft law’ such as guidelines, notices or communications.^48^ In its classical understanding, communications include policy evaluations, commentaries or explanations of action-programmes or brief outlines on future policies.^49^ Although acts of soft-law are not binding for individuals, it follows from the case law that they may nevertheless produce certain legal effects. Indeed, the provisions of acts of ‘soft law’ are, by virtue of the duty of sincere cooperation enshrined in Article 4(3) TEU, and are to be taken into due account by the Member States’ authorities.^50^ Yet, as noted by Advocate General Wahl, ‘that duty cannot be understood as making those rules binding — not even de facto — on pain of eluding the legislative procedure set out in the FEU Treaty’.^51^ The EGD is thus clearly a policy tool to be translated into the legal measures, but anchored in the duty of sincere cooperation.

More importantly, EGD as a response to climate change and an instrument of a change for the Union should be seen against the background of classical sources of EU environmental law. Although seemingly one cannot notice any particular divergence compared to other fields of EU law, two sources merit a special attention in EU environmental law.^52^ First, environmental action programmes which are the cornerstone of the EU environmental policy since its origins, have, contrary to other fields of EU law, a particular role and legal status. Pursuant to Article 192(3) TFEU, the general action programmes are adopted under the ordinary legislative procedure. Naturally, they are legally binding for the EU institutions. As a consequence, the environmental action programmes are not soft law, but hard law from a legal point of view.^53^ From the ambitious perspective of the EGD, the environmental action programme could have been thus an efficient option to translate the ambition of the climate neutrality by 2050. Secondly, environmental principles in the meaning of Article 191(2) TFEU.^54^ They have to be respected by the EU institutions in the context of EU environmental policy and also by Member States when implementing EU law. Principles of precaution, prevention, of rectification at source and polluter-pays principle have to be observed when adopting EU secondary law. Although their legal status through the angle of enforceability remains uncertain,^55^ they perform a relevant role in respect of multi-dimensional and scientifically complex environmental problems.^56^ Environmental principles carry a message understood as a particular legal commitment to environmental protection and sustainability. While reading through the Commission’s communication on the EGD no trace of a reference to the classical environmental principles can be found. Only sustainability has gained a particular attention as an underlying concept, but with a particular emphasis for its economic potential. Yet, in a larger perspective and certainly at the stage of turning EGD’s objectives into the legal acts, environmental principles should demonstrate their legal meaning. Finally, one of the critical environmental concepts enshrined in Article 11 TFEU, principle of environmental integration is not referred to in the EGD which illustrates a critical dissonance between the EGD as an ambitious policy measure and the legacy and relevance of the EU environmental law for the purpose of its implementation.

Interestingly, the EGD communication also refers to a new policy principle – “green oath: do no harm”. While indicating the need for all the “EU actions and policies to support a successful and just transition towards a sustainable future”, the Commission proposes a relatively ambiguous and programmatic “do no harm” principle.^57^ In order to ensure that all EGD initiatives achieve their objectives in the most effective way and all other EU initiatives comply with a green oath to ‘do no harm’, the scope and meaning of the no harm concept is to be translated at the level of particular legislative proposals. Yet, the “no harm” seems to amount to the policy commitment according to which in all its actions the Union should avoid doing harm to environment, commitment which is far too programmatic to grasp its substance from the enforceability perspective. Likewise, it does not seem to rely on the EU primary or secondary law references.^58^ In its proposal of the European Climate Law, which is based on Article 191(1) TFEU the Commission refers to the green oath, without further explanation of its meaning.^59^ It is to be expected that in areas in which the Commission intends to propose new legislation in 2021 to align them to the new 50/55% climate targets for 2030, the ‘no harm’ principle will be more explicitly substantiated.^60^ The scope of the ‘green oath’ seems to be more comprehensible from the perspective of the EU funding based notably on the recent taxonomy regulation.^61^ Likewise, according to the European Council conclusions of July 2021, “EU expenditure should be consistent with Paris Agreement objectives and the “do no harm” principle of the European Green Deal”.^62^

Fundamentally, what the EGD fails to address and explore is the constitutional dimension of environmental protection in the EU legal order. Constitutional entrenchment of environmental protection seems to be precisely a missing point of the EGD. Notwithstanding the EGD being considered “Europe’s man on the moon moment…”,^63^ it remains a program, a roadmap of highly technical announcements of new legislative and political initiatives, which are meant to lead to the new prosperity and growth and ambitious climate targets. The Commission is consequently proposing or going to propose an impressive number of legal acts. It implies unprecedented efforts at the EU institutional level and implementation efficiency at the Member States’ level. Likewise, undoubtedly, climate neutrality entails costs at the social and economic levels. The key element is the concept of the transition or transformation which shall bridge the current legal and policy context and the 2050 economic status quo of the Union as a global leader. It requires thus a unique engagement of the public and private investors as well as of the civic society and of each and every EU citizen, conscient of the risk of non-action towards the climate change. In the author’s view, the major enabling components which should be relied upon in order to consolidate all of these ambitions are the multidimensional principle of solidarity and the values underlying the ‘Union’s constitutional core’ in the light of Article 2 TEU. To conclude, it may be question whether given its nature and its content the EGD does truly represent a ‘constitutional moment’^64^ of environmental protection in the EU legal order. Hopefully, its full legal potential is still to unfold.

## Financial dimension of the EGD – from environmental sustainability ambitions to the post pandemic responses capacities

### Between macroeconomics and financial responses to COVID-19 pandemics

As announced by the Commission, meeting the ambitious objectives of the EGD entails investments.^65^ Indeed, what EGD demonstrates particularly well is to what extent environmental protection and climate change related commitments require investments and new rationale based on the concept of sustainable finance. Thus, the EGD can only be seen jointly with the EGD Investment Plan. Financial and economic dimension of the EGD is of outmost importance to make EU regulatory framework efficient tool of climate neutrality.

From the constitutional angle, in Article 3 TEU the treaties establish a balance between various objectives such as sustainable development, high level of environmental protection and internal market perspective, economic growth and social considerations. The EGD must thus find its own place in the framework of balancing of the economic and non-economic objectives of the Union. In particular, in year 2020, the COVID-19 pandemic has irreversibly affected the Union’s economic viability. The unprecedented measures in the field of the cohesion policy,^66^ EU budgetary instruments such as New Generation EU (NGEU),^67^ EU Recovery Instrument,^68^ amendments of the system of own resources^69^ show that tangible crisis may trigger tangible financial responses at the level of the Union. Yet, the political and legal mobilization following the COVID-19 economic shock can only bring its results if coupled with measures respecting environmental sustainability driven by the climate protection approach.

At the macro-economic level, the EGD aims at re-focusing of the European Semester which is a non-binding framework for the coordination of economic and fiscal policies across the Union. It should be noted that reshaping the European Semester via the measures adopted as a follow-up of EGD must remain in harmony with various and often competing objectives of the Union as set in Article 3 TEU. EGD seems to address this challenge by focusing on the “new growth strategy that aims to transform the EU into a fair and prosperous society, with a modern, resource-efficient and competitive economy where there are no net emissions of greenhouse gases in 2050 and where economic growth is decoupled from resource use.”.^70^ In May 2020, the Commission issued the country specific recommendations which included clear references to both pandemic and climate change challenges.^71^ The Commission thus, via this coordination instrument, underlines four dimensions of the so-called “competitive sustainability” as presented in the Annual Sustainable Growth Strategy which is at the heart of Europe’s social market economy: “economic stability, social fairness, environmental sustainability and productivity and competitiveness, as well as place specific emphasis on health”.^72^ In its Communication, the Commission rightly emphasized the substantive link between the post-pandemic recovery strategy and the green and digital transition in line with the EGD. Indeed, competitive sustainability arises to the core of an economic agenda allowing EU and its Member States to achieve the UN Sustainable Development Goals (UNSDGs) in joint efforts of reaching climate neutrality by 2050. In this regard, it is rightly argued that ‘A key question for the successful integration of the environmental sustainability leg of the SDGs in the economic policy of the new European Semester will be the choice of indicators.”^73^

One of the ways the EGD intends to reach the 2050 objectives is through financial measures. Effectiveness of the EGD will be consequently measured against its capacity to mobilise climate dedicated funding. The EGD intends to address this financial dimension, among others, through investments, mobilizing private, public funds, dedicating a part of the EU budget to climate action, providing support via the European Investment Bank. The Commission adopted the EGDIP which constitutes the investment pillar of the EGD. According to the Commission, the Investment Plan was meant to raise EU funding and create an enabling framework to *facilitate* and *stimulate* the public and private investments needed for the transition to a climate-neutral, green, competitive and inclusive economy. The natural candidate to fulfill these ambitions is the EU cohesion policy and Article 174 TFEU, triggering the co-financing by the Member States. The EGDIP is based on three dimensions. *Financing* refers to mobilising at least €1 trillion of sustainable investments over the next decade. According to the Commission a “greater share of spending on climate and environmental action from the EU budget than ever before will crowd in private funding, with a key role to be played by the European Investment Bank”. Under the *enabling* component, the Commission intended to provide incentives to unlock and redirect public and private investment. Finally, by *practical support* the Commission indented to provide support to public authorities and project promoters in planning, designing and executing sustainable projects. The new climate targets permeates nowadays the new headings of EU spending under Multiannual Financial Framework (MFF), in particular in relation to the environment and cohesion.^74^ The flagship instrument of the EGDIP is the Just Transition Mechanism (JTM). According to the Commission, the JTM is ‘a key tool to ensure that the transition towards a climate-neutral economy happens in a fair way, leaving no one behind.’^75^

### The key – sustainable finance

Before addressing the details of major financial measures aimed at achieving the green transition as announced in the EGDIP, one may ask whether the reference of sustainability which underlies the EGD focuses on environmental component? Does sustainable mean greener in the framework of the EGD? The concept or principle of sustainable development is codified in the EU primary law in Article 3(3) TEU and amounts to the constitutional commitment of the Union. Naturally, its legal status and substance are marked by great complexity stemming from the very nature of sustainable development which covers three-fold dimension: economic, environmental and social. Furthermore, sustainable development is closely related and enhanced by the EU principle of environmental integration enshrined in Article 11 TFEU. This particular relationship is not explicitly addressed in the EGD. It is nevertheless interesting to see whether EGD as such is liable to affect the three-fold nature of sustainable development. Would the Union via this ambitious communication attempt to re-balance the concept of sustainable development while fully committing to the UNSDGs? One of the major fields where this re-balancing is taking place is precisely financial and economic measures. The new key terms for the efficient EGD are sustainable finance and sustainable investment. In particular, it follows from the Commission’s action plan on financing sustainable growth^76^ that public and private investment are needed to transform the EU economy to deliver on climate, environmental and social sustainability goals.^77^

Sustainable finance is considered as key aspect of the EGD. It reflects the idea of making sustainability considerations part of financial decision-making process. Sustainable finance means ‘taking due account of *environmental* and *social and governance* considerations when making investment decisions’.^78^ A particular example of an environmentally orientated funding approach is illustrated by the concept of *sustainable investment* enshrined in the recently adopted taxonomy regulation.^79^ The taxonomy regulation is intended to provide with a common framework identifying to what degree economic activities may be considered ‘environmentally sustainable’.^80^ The EU legislator adopted thus an exhaustive list of six environmental objectives which cover climate change mitigation, climate change adaptation, sustainable use and protection of water and marine resources, transition to a circular economy, pollution prevention and control, as well as protection and restoration of biodiversity and ecosystems. An ‘environmentally sustainable investment’ has been thus given, under the taxonomy regulation, a legally binding definition.^81^ While referring to the ‘no harm’ concept, taxonomy regulation provides in its preamble that, ‘an economic activity should not qualify as environmentally sustainable if it causes more harm to the environment than the benefits it brings.’^82^

Nevertheless, the flagship proposal indented to address the transition towards climate neutrality in the Union is Just Transition Mechanism. JTM is intended to provide support to territories facing serious socioeconomic challenges related to the transition towards climate neutrality. JTM is based on three pillars: Just Transition Fund (JTF),^83^ InvestEU^84^ and Public Sector Loan Facility.^85^

Based on the cohesion legal basis, the JTF proposed in January 2020 as a new EU structural fund was intended “to alleviate the impact of the transition by financing the diversification and modernization of the local economy and by mitigating the negative repercussions for employment”. JTF was meant to provide support to territories facing serious socio-economic challenges deriving from the transition process towards the climate-neutral economy of the Union by 2050.^86^ Following the recent developments related to the COVID-19 crisis, the Commission proposed in May 2020 amendments aiming, notably, at extending financing sources of the JTF to external assigned revenue stemming from the European Recovery Instrument.^87^ Following these developments the JTF budget has considerably increased, at this stage, to €40 billion,^88^ pending further negotiations with the European Parliament. As announced in the European Council conclusions, JTF relies on the climate conditionality based on the commitment to a national objective of climate neutrality by 2050.^89^ In case such commitment is not accepted by the Member State, access to JTF might be limited to 50% of the national allocation.^90^

Secondly, a dedicated Just Transition Scheme has been proposed to be established under InvestEU, as the second pillar of the JTM.^91^ InvestEU Fund consists of an EU budget guarantee for the financial products provided by the implementing partners. Although the Commission’s communication mentioned initially that the negotiations between the European Council and the Parliament on InvestEU, which led to an agreement in April 2019, would not be re-opened, in May 2020, the Commission withdrew its earlier proposal presented for the InvestEU Programme and presented a new legal instrument in order ‘to reflect the specific post-pandemic needs of the European economy’.^92^ InvestEU was also specifically addressed in the July 2020 conclusions of the European Council: ‘its overall objective is to support the policy objectives of the Union by mobilising public and private investment within the EU that fulfil the criterion of additionality, thereby addressing market failures and sub-optimal investment situations that hamper the achievement of EU goals regarding sustainability, competitiveness and inclusive growth.’^93^

The third pillar of the JTM consisted in creation of a public sector loan facility at the European Investment Bank (EIB), partly guaranteed by the EU budget, to mobilise between €25 billion to €30 billion of additional public investments in 2021-2027.^94^ It should be noted that projects which are not eligible under the JTF could be financed under other JTM components. Yet, since the legislative process is still ongoing, it is not possible to determine precisely the respective scopes of the discussed pillars of the JTM. The European Parliament may thus become a crucial actor of the green transition in many pending files related to the climate agenda.

## Conclusion

F. Sherwood Rowland, a chemist who shared a Nobel Prize for the discovery of the ozone hole, famously observed that ‘(t)he work is going well, but it looks like it might be the end of the world’.^95^ One should hope that the EGD will not be just “work going well”, but will initiate new substantive *attitude* towards environmental and climate considerations at the political, institutional, regulatory, administrative and individual levels. EGD will undoubtedly trigger indirect or direct financial impact on all areas of the EU economy and industry.

Yet, the green transition and its financing can only happen where it involves both the EU and State actors and the EU citizens who will agree to bear the costs and participate in the process, notably in the framework of the announced Climate Pact. The enhanced solidarity of the green transition towards climate neutrality should be a synonym of the European Green Deal.

## Footnotes

1 https://www.nytimes.com/interactive/2020/climate/climate-change-books.html.

2 https://www.unenvironment.org/resources/emissions-gap-report-2019 and https://www.unenvironment.org/resources/emissions-gap-report-2019.

3 https://www.theguardian.com/commentisfree/2020/aug/19/climate-crisis-leaders-greta-thunberg; https://www.extinctionrebellion.be/en/.

4 https://www.climatechangenews.com/2020/04/09/european-green-deal-must-central-resilient-recovery-covid-19/; https://www.euractiv.com/section/climate-environment/news/timmermans-defends-higher-eu-goals-on-climate-change/.

5 See, European Council conclusions of 17-21 July 2020, available at: https://www.consilium.europa.eu/media/45109/210720-euco-final-conclusions-en.pdf, point 21.A.

6 *Jääskinen*, [1].

7 Communication from the Commission to the European Parliament, the European Council, the Council, the European Economic and Social Committee and the Committee of the Regions, The European Green Deal, COM(2019) 640 final.

8 COM(2019) 640 final, annex: (1) Climate ambition (2) Clean, affordable and secure energy (3) Industrial strategy for a clean and circular economy (4) Sustainable and smart mobility (5) Greening the Common Agricultural Policy/ “Farm to Fork strategy” (6) Preserving and protecting biodiversity (7) Towards a zero-pollution ambition for a toxic free environment (8) Mainstreaming sustainability in all EU policies (9) The EU as a global leader (10) Working together-a European Climate Pact.

9 COM(2019) 640 final, p. 2.

10 *Miccinilli*, [2], p. 15.

11 *Chalmers*, [3]; *Scott*, [4].

12 Paris Agreement (Dec. 13, 2015), in UNFCCC, COP Report No. 21, Addendum, at 21, U.N. Doc. FCCC/CP/2015/10/Add, 1 (Jan. 29, 2016) (Paris Agreement). The EU adhered to the Paris Agreement, see Decision 2016/1841on the conclusion, on behalf of the European Union, of the Paris Agreement adopted under the United Nations Framework Convention on Climate Change, (OJ 2016, L 282, p. 1).

13 https://sustainabledevelopment.un.org/post2015/transformingourworld.

14 https://ec.europa.eu/environment/sustainable-development/SDGs/index_en.htm.

15 Communication from the Commission to the European Parliament, the European Council, the Council, the European Economic and Social Committee and the Committee of the Regions, Sustainable Europe Investment Plan, European Green Deal Investment Plan, COM(2020) 21 final.

16 https://ec.europa.eu/info/publications/200114-european-green-deal-investment-plan_en, https://ec.europa.eu/commission/presscorner/detail/en/qanda_20_24.

17 *Siddi*, [5], p. 4.

18 Communication from the Commission to the European Parliament, the European Council, the Council, the European Economic and Social Committee and the Committee of the Regions, A new Industrial Strategy for a globally competitive, green and digital Europe, COM (2020) 102 final.

19 Directive 2000/60/EC of the European Parliament and of the Council of 23 October 2000 establishing a framework for Community action in the field of water policy, (OJ L 327, 22.12.2000, p. 1–73).

20 Case C-461/13, *Bund für Umwelt und Naturschutz Deutschland*, EU:C:2015:433, para 50.

21 For further reading, see *Sikora* [7], p. 2-15.

22 About the concept of ‘environment’, see, inter alia, *Kiss*, *Shelton*, [6], p. 4.

23 *May*, *Daly*, [7], p. 91.

24 First Communication of the Commission about the Community’s policy on the environment, SEC (71) 2616 final, Brussels 22.7.1971.

25 *Jans*, *Vedder*, [8], p. 3-13; *Kramer*, [9], p. 1-5; N. *Haigh* [10], p. 64-76.

26 *Winter*, [11], p. 38-47.

27 Judgement in Case 240/83, *Procureur de la République v Association de défense des brûleurs d’huiles usagées* (*ADBHU*), EU:C:1985:59.

28 *Jacobs*, [12], p. 185-205.

29 *von Homeyer*, [13], pp. 1-26.

30 *Hayward* [14], p. 129-130.

31 *Kloepfer*, [15], p. 112-115.

32 *Singh Ghaleigh*, [16], p. 419-462.

33 *Sikora*, [17], p. 57. Whether climate change is considered as an autonomous branch of EU law or rather an off-spring of EU environmental law, see *Hilson* [18].

34 M. Humphreys, [19]. Sustainable Development in the European Union: A General Principle, Abigdon 2018, p. 44.

35 *Jans*, *Vedder*, [8], p. 41.

36 *Missonne*, [20].

37 *Sikora*, [17].

38 See Opinion of AG Bot in Case C-195/12, *IBV & CIE*, EU:C:2013:293, point 82.

39 See Opinion of AG Sharpston in Case C-557/15 *Commission v Malta*, EU:C:2017:613, point 44; and in Case C-664/15, *Protect Natur-, Arten- und Landschaftsschutz Umweltorganisation*, EU:C:2017:760, point 68.

40 See Opinion AG Kokott in Case C-444/15, *Associazione Italia Nostra Onlus*, EU:C:2016:665, point 25.

41 See Opinion of AG Kokot in Case C-723/17, *Craeynest and Others*, EU:C:2019:168, point 53.

42 Judgement in Case C-723/17 *Craeynest and Others*, EU:C:2019:533, para 33; judgement in Case C-129/16, *Túrkevei Tejtermelő Kft*., EU:C:2017:547, para 33.

43 Opinion of AG Bot in joined Cases C-643/15 and C-647/15, *Slovakia and Hungary v Council*, EU:C:2017:618, point 17.

44 Regulation (EU) 2018/842 of the European Parliament and of the Council of 30 May 2018 on binding annual greenhouse gas emission reductions by Member States from 2021 to 2030 contributing to climate action to meet commitments under the Paris Agreement and amending Regulation (EU) No 525/2013, (OJ L 156, 19.6.2018, p. 26–42).

45 Ex multis: *Kramer*, [21], *Thieffry* [23], *Siddi* [5], *Binet*, [22], *Miccinilli*, [3], *Petit* [24], *Hodne* [25], *Eyl-Mazzega*, *Matthieu* [26].

46 COM(2019) 640 final.

47 *Eyl-Mazzega*, *Matthieu* [26].

48 Judgments in joined Cases C-75/05 P and C-80/05 P, *Germany and Others v Kronofrance*, EU:C:2008:482, paras 60 and 61 and C-387/97, *Commission v Greece*, EU:C:2000:356, para 87.

49 Cailles, Ruffert, Art. 288 (ex-Art. 249 EGV) [Rechtsakte des Unionsrechts] Ruffert in Calliess/Ruffert | AEUV Art. 288 Rn. 1-108 | 5. Auflage 2016, beckonline.de.

50 Judgment in Case C-322/88, *Grimaldi*, EU:C:1989:646, paras 18 and 19; and Opinion of AG Kokott in Case C-226/11, *Expedia*, EU:C:2012:544, point 38.

51 Opinion of AG Wahl in case C-526/14 *Kotnik*, EU:C:2016:102, point 38.

52 *Epiney*, [27].

53 Under Article 192(3) TFEU, the Parliament and Council adopt the general action programmes setting out the priority objectives to be attained. Subsequently, measures necessary for the implementation of the environmental action programme are to be adopted on the basis of Article 192(1) TFEU and (2) or on a different relevant legal basis in the TFEU. In particular, given the transversal nature of environmental considerations, measures referring to environmental policy may be part of other policies.

54 *De Sadeleer*, [28].

55 *Scotford*, [29].

56 *De Sadeleer*, [28].

57 COM (2019) 640 final, point 2.2.5.

58 For example, the principle of ‘do no significant harm’ is referred to in Regulation (EU) 2019/2088.

59 Proposal for a Regulation of the European Parliament and of the Council establishing the framework for achieving climate neutrality and amending Regulation (EU) 2018/1999 (European Climate Law), COM(2020) 80 final.

60 The Commission announced the following measures to be amended: The directive on the greenhouse gas emissions trading scheme, the effort sharing regulation, the regulation on land use, the energy efficiency directive, the directive on renewable sources of energy, the CO2 emission performance standards for cars and vans.

61 Regulation (EU) 2020/852 of the European Parliament and of the Council of 18 June 2020 on the establishment of a framework to facilitate sustainable investment, and amending Regulation (EU) 2019/2088.

62 https://www.consilium.europa.eu/media/45109/210720-euco-final-conclusions-en.pdf.

63 https://ec.europa.eu/commission/presscorner/detail/en/speech_19_6749.

64 Allot, [30], p. 489.

65 https://ec.europa.eu/commission/presscorner/detail/en/IP_19_6691.

66 https://ec.europa.eu/regional_policy/en/newsroom/coronavirus-response. See, Coronavirus Response Investment Initiative (CRII) and Coronavirus Response Investment Initiative Plus (CRII+).

67 For further reading see https://ec.europa.eu/info/live-work-travel-eu/health/coronavirus-response/recovery-plan-europe/pillars-next-generation-eu_en; https://www.europarl.europa.eu/RegData/etudes/BRIE/2020/651993/EPRS_BRI(2020)651993_EN.pdf.

68 Proposal for a COUNCIL REGULATION establishing a European Union Recovery Instrument to support the recovery in the aftermath of the COVID-19 pandemic, COM/2020/441 final.

69 See amended proposal for a Council decision on the system of Own Resources of the European Union, COM(2020) 445 final.

70 COM(2019), 640 final, point 1.

71 Communication from the Commission to the European Council the Council, the European Central Bank, the European Economic and Social Committee, the Committee of the Regions, and the European Investment Bank, COM(2020) 500 final.

72 COM(2000) 500 final, p. 1.

73 See, Delivering Green Deal - the role of a reformed European Semester within a new sustainable economy strategy, Institute for Environmental European Policy, available at https://ieep.eu/uploads/articles/attachments/fa7f2aa6-35d3-4fa2-88b4-98e1e79356e5/Delivering%20the%20Green%20Deal%20through%20the%20European%20Semester%20-%20Final_compressed.pdf?v=63749848881, p. 13.

74 In the MFF context, most relevant from the climate change perspective are Heading 3 “Natural resources and environment” and Heading 2 “Cohesion and values”. Heading 3 covers main financial instruments for the common agricultural policy (CAP) such the European Agricultural Guarantee Fund (EAGF) and the Agricultural Fund for Rural Development (EAFRD).

75 https://ec.europa.eu/info/strategy/priorities-2019-2024/european-green-deal/actions-being-taken-eu/just-transition-mechanism_en.

76 Communication from the Commission to the European Parliament, the European Council, the Council, the European Central Bank, the European Economic and Social Committee and the Committee of the Regions, Action Plan: Financing Sustainable Growth, COM/2018/097 final. Action Plan, COM(2018) 97, see, F. Möslein, K. Engsig Sørensen, The Commission’s Action Plan for Financing Sustainable Growth and its Corporate Governance Implications, 2018, Nordic & European Company Law Working Paper No. 18-17, available at SSRN: https://ssrn.com/abstract=3251731.

77 For further reading, see https://ec.europa.eu/info/publications/sustainable-finance-renewed-strategy_en.

78 https://ec.europa.eu/info/business-economy-euro/banking-and-finance/sustainable-finance/what-sustainable-finance_en.

79 Regulation (EU) 2020/852 of the European Parliament and of the Council of 18 June 2020 on the establishment of a framework to facilitate sustainable investment, and amending Regulation (EU) 2019/2088, (OJ L 198, 22.6.2020, p. 13–43).

80 In accordance with its Article 1, taxonomy regulation ‘establishes the criteria for determining whether an economic activity qualifies as environmentally sustainable for the purposes of establishing the degree to which an investment is environmentally sustainable’.

81 Taxonomy regulation has nevertheless left aside issues such as nuclear energy and its relevance for environmentally orientated investments.

82 Recital 40 of the taxonomy regulation.

83 Proposal for a Regulation of the European Parliament and of the Council establishing the Just Transition Fund, COM(2020)22 final together with amendment of the Common Provisions Regulation, see COM(2020) 23 final.

84 Proposal for a Regulation of the European Parliament and of the Council establishing the InvestEU Programme, COM/2018/439 final.

85 Proposal for a Regulation of the European Parliament and of the Council on the public sector loan facility under the Just Transition Mechanism COM/2020/453 final.

86 Article 1 of the Proposal for a Regulation of the European Parliament and of the Council establishing the Just Transition Fund, COM(2020)22 final.

87 Amended proposal for a Regulation of the European Parliament and of the Council establishing the Just Transition Fund, COM/2020/460 final.

88 https://www.bruegel.org/2020/06/one-last-push-is-needed-to-improve-the-just-transition-fund-proposal/.

89 See, European Council conclusions of 17-21 July 2020, available at: https://www.consilium.europa.eu/media/45109/210720-euco-final-conclusions-en.pdf.

90 See, Council Conclusions of 19 December 2019, available at https://www.consilium.europa.eu/media/41768/12-euco-final-conclusions-en.pdf. Although Poland was not mentioned, this conditionality was designed to address situation of this Member State.

91 COM (2018)439 final.

92 COM(2020) 403 final.

93 European Council conclusions of 17-21 July 2020, available at: https://www.consilium.europa.eu/media/45109/210720-euco-final-conclusions-en.pdf, point 30.

94 COM/2020/453 final.

95 *Sands*, [31], p. 7.
